# Understanding malaria treatment patronage from informal healthcare providers in Nigerian urban settlements: insights from community members and providers

**DOI:** 10.1186/s12936-025-05255-3

**Published:** 2025-01-23

**Authors:** Eniola A Bamgboye, Akintayo Olamide Ogunwale, Adamu Al-Mukhtar, Bello Musa, Laurette Mhlanga, Morenikeji Olawuwo, Adeniyi Fagbamigbe, Joshua Akinyemi, IkeOluwapo Ajayi, Ifeoma D Ozodiegwu

**Affiliations:** 1 Department of Health Informatics and Data Science, Loyola University Chicago, Chicago, IL USA; 2 Epidemiology and Biostatistics Research Unit, Institute for Advanced Medical Research and Training (IAMRAT), College of Medicine, University of Ibadan, Ibadan, Nigeria; 3 Department of Public Health, Bowen University, Iwo, Nigeria; 4 Department of Medical Microbiology and Parasitology, Bayero University, Kano, Nigeria; 5 Department of Community Medicine, Bayero University, Kano, Nigeria; 6 Department of Epidemiology and Medical Statistics, University of Ibadan, Ibadan, Nigeria

**Keywords:** Urban malaria, Treatment, Traditional healers, Patent proprietary medicine vendors, Patronage

## Abstract

**Background:**

Informal Healthcare Providers (IHCPs), including Proprietary Patent Medicine Vendors (PPMVs), drug peddlers, traditional healers, and herbal drug sellers are often the first choice for malaria treatment, especially in urban slums. Unplanned urbanization significantly impacts malaria transmission by creating cities with inadequate safety nets and healthcare access, increasing reliance on IHCPs. While the World Health Organization recognizes IHCP’s crucial role and emphasizes integrating them into formal healthcare for improved malaria care, they lack requisite training in malaria management and operate outside official regulations, raising concerns about the quality of care they provide. Understanding IHCPs' perceptions and practices is essential for their proper integration. This study explored the perceived malaria burden, IHCPs’ competence in malaria treatment, and reasons for visiting IHCPs in various urban settlements from both community member and provider perspectives.

**Methods:**

This qualitative cross-sectional study was carried out in Ibadan and Kano metropolises. Eighteen Focus Group Discussions among 157 adult community members and twelve Key-Informant Interviews among PPMVs, drug peddlers, traditional healers and herbal drug sellers were conducted in these cities. Participants were drawn purposively from settlements—designated as formal, informal, and slum based on local definitions—in selected wards within the cities. Data were collected using pre-tested guides and analysed thematically.

**Results:**

This study reveals that malaria remains a significant health problem in these Nigerian cities. Patronage of IHCPs generally is driven by affordable treatment, perceived mildness of illness, and access to credit facilities. However, cultural belief was key to patronage of traditional healers and herbal drug sellers, largely among informal and slum residents. Furthermore, while IHCPs had a strong perceived competence in managing malaria cases, inadequate diagnosis and treatment were standard practices.

**Conclusions:**

IHCPs remain consistently patronized across urban settlements. Educating and equipping IHCPs with diagnostic tools, enhancing access to affordable healthcare, and raising public awareness is crucial for proper malaria management and promoting collaborations with formal healthcare providers.

## Background

The World Health Organization (WHO) aims to reduce malaria incidence and mortality by 90% and eliminate malaria in at least 35 countries by 2030. To achieve this, one of the central pillars of the Global Technical Strategy for Malaria (2016–2030) is to ensure universal access to diagnosis and prompt, effective treatment of malaria in public and private health facilities, as well as at the community level [1, 2]. However, according to the 2023 Malaria Report, approximately one-third of febrile children in sub-Saharan Africa do not seek treatment at a health facility [2]. Among those who did seek treatment, only about half received a proper diagnosis, highlighting the slow progress towards achieving the targets set by the WHO [2].

Informal Healthcare Providers (IHCPs) are diverse practitioners who provide healthcare services without formal medical training. They include Patent Proprietary Medicine Vendors (PPMVs), drug peddlers, traditional healers and herbal drug sellers, and are often the first point of contact for malaria treatment in sub-Saharan African countries [3]. Due to their widespread public use, the WHO recognizes IHCPs as crucial healthcare providers who need to be integrated into the formal healthcare system in such countries to improve service delivery in areas such as malaria management [4]. However, most IHCPs lack professional and requisite training in malaria management, raising concerns about the quality of their practices [5].

Nigeria has one of the lowest rates of formal healthcare-seeking behaviour for suspected malaria in children under five, with less than 30% of febrile children being taken to formal healthcare facilities for consultation and testing [3]. Instead, IHCPs, especially PPMVs, are the most common source of anti-malarial drugs and treatment for fever or suspected malaria, even in cities [6, 7]. Cities are characterized by spatial variation in factors such as the social and physical environment, as well as access to healthcare and other services [8]. These variations contribute to the emergence of distinct settlement types—formal, informal, and urban slums- which can also influence healthcare-seeking behaviours for diseases like malaria [9].

The increase in informal settlements and slums within Nigerian cities, likely due to unplanned rapid urbanization, suggests that patronage of IHCPs may be sustained or even rise [10]. Recent studies in Nigerian cities have also found that community members especially those from informal settlements and slums often seek malaria treatment from IHCPs [11, 12].

Several reasons have been identified for this practice in Nigeria [13–15]. Studies have reported that community members often turn to traditional healers, especially when illnesses persist after conventional treatment, due to a belief in the superior efficacy of traditional or herbal medicine [13, 14]. Additionally, people prefer PPMVs for treatment of suspected malaria because of the affordability of drugs, availability of preferred medications, shorter waiting times, and more hospitable service [15]. These studies bring to fore the continuous patronage of IHCPs, even in Nigerian cities.

Given the crucial role IHCPs play, understanding their malaria management practices is essential for effective integration into the formal health system. Many IHCPs learn through apprenticeship and operate outside official regulations, highlighting the need to investigate their practices [16].

This study drew on the Health Belief Model, which suggests that health-seeking behaviour is influenced by factors such as perceived susceptibility, severity, benefits, barriers, self-efficacy, and cues to action [17]. Easy accessibility and interaction with IHCPs in communities can act as cues, influencing the choice between formal and informal healthcare providers.

While some studies [18–20] have assessed community members' use of IHCP services, few have explored the variations across urban settlement types and triangulated findings with the perspectives of the providers. This dual perspective can provide valuable insights to guide the effective integration of IHCPs into the formal healthcare system, to enhance malaria service delivery.

This study explored the perceived burden and causes of malaria, IHCPs' competence in treating malaria, and the drivers of community members’ patronage of IHCPs in different urban settlement types in two Nigerian cities.

## Methods

This study was conducted between January and February 2023 in two Nigerian mega-cities namely Ibadan and Kano.

### Context

These cities were purposively selected in collaboration with the Nigeria National Malaria Elimination Programme (NMEP) based on the relatively high burden of malaria and the significant investment in malaria control programmes. Ibadan, the capital city of Oyo State, is Nigeria’s third-largest city located in the South-West geo-political zone. Kano is the largest cosmopolitan city and the capital of Kano State in the North-West geo-political zone of Nigeria. In 2022, the Ibadan metropolis area had a population of 1,009, 123, while Kano metro had a population of 3,196,510 [21]. The healthcare system in these cities covers both formal and informal health services. The formal health system is organized into three tiers: primary, secondary, and tertiary.

Ibadan metropolis has 301 Primary facilities, 15 Secondary and 2 Tertiary hospitals, while Kano metropolis has 191 Primary facilities, 8 Secondary and 1 Tertiary hospital. The cities are also served by private and specialist health facilities [22]. However, most malaria-related services are readily provided at primary health facilities. The cities also have various IHCPs, including PPMVs, drug peddlers, traditional healers and herbal drug sellers, who offer different health care services, including malaria management.

### Study design and sampling

This was an exploratory cross-sectional study employing Focus Group Discussions (FGDs) and Key Informant Interviews (KIIs). Participants for this study were drawn from four (4) of fifty-nine (59) and five (5) of sixty-six (66) wards in the Ibadan and Kano metropolis, respectively. The wards were selected using a model-based clustering algorithm, which used environmental, settlement characteristics, and population-dependent malaria risk indices [23]. In each ward, the settlements were classified as formal, informal, and slums based on the findings of multi-stakeholder dialogues (MSDs) held in the two cities. The details of this process have been published elsewhere [23].

In this study, the settlements are defined as follows:

1. Formal settlements were conceptualized as well-planned layout areas or government residential areas not densely populated. They are characterized by the availability of social amenities and infrastructure including good roads, electricity, good drainage systems, schools, hospitals and police stations.
2. Informal settlements were considered as areas not well developed, basically developed by the community and not by the government consisting of mixed types of houses not following any government regulations. They are characterized by inadequate social amenities and infrastructure.
3. Slums were operationally defined as settlements that are unplanned and have abysmal or poor housing conditions, with no or limited basic amenities and usually densely populated and inhabited by people of low socio-economic status.

Participants were then subsequently recruited by purposive sampling in the three settlement types with the help of community gatekeepers such as Ward Development Committee Chairmen, community leaders, heads of local associations who were identified during the MSDs.

Eighteen FGDs involving 157 participants across the three different settlement types were conducted in both cities. The FGD participants were community members aged 18 years and above. In each city, FGD groups were stratified into male, female, and mothers of under-five children, and three FGD sessions were conducted in each group, respectively. This was done to better understand the perspectives and diverse viewpoints of different groups on their patronage of IHCPs for malaria treatment. Each FGD group consisted of an average of eight discussants.

To understand provider perspectives, 12 Key Informant Interviews (KIIs) were conducted among four PPMVs, three drug peddlers, two traditional healers, and three herbal drug sellers,in both cities. These participants were also recruited with the help of the community gatekeepers. The detailed characteristics of the participants are presented in Appendices 1 and 2.

### Data collection

Validated FGD and KII guides were used for data collection. Content and face validation of the guides were conducted by members of the study team who are experts in malaria epidemiology and qualitative research methods. The FGD and KII guides were designed to assess the perceived burden of malaria by asking participants about how common the cases of malaria in their community were, the groups of people most affected, and the key factors driving transmission in their community. Regarding competence, FGD participants who had visited any of these IHCPs were asked to share their experiences when seeking malaria treatment, while KII participants were asked to assess their own ability to treat malaria effectively. Additionally, participants were asked about the reasons community members choose to patronize these IHCPs.

Data were collected by trained research assistants, who had previous experience in qualitative research and were proficient in both English and the local language in each study site (Yoruba -for Ibadan; Hausa- for Kano). The research assistants were exposed to a two-day training focused on relevant issues, including the content of the interview guides, interviewing, probing, recording skills, and data transcription. Following the training, pre-tests comprising three FGDs sessions (one per settlement type) and four KIIs (one in each category—PPMVs, drug peddlers, traditional healers and herbal drug sellers) were conducted in a ward with a diverse mix of formal, informal and slum settlements that was not selected for the larger survey in both cities. The pre-testing helped to improve the FGD and KII guides and sensitize the RAs on the issues that might arise during the data collection. Following the pre-testing, the FGD and KII guides were revised with inputs from subject matter experts to enhance the clarity of ambiguous questions.

The instruments were originally designed in English and later forward-back translated into Yoruba language (the local dialect in Ibadan) and Hausa language (the local dialect in Kano) for ease of understanding and administration.

A team of two trained RAs comprising a moderator, and a note-taker conducted the KIIs and FGDs. The KIIs and FGDs were conducted in quiet, comfortable settings considered confidential and safe by the participants. All the KIIs and FGDs were audiotaped, and field notes were taken. The average time to complete the KIIs and FGDs was 35 and 55 min, respectively. Interviews were conducted primarily in Yoruba and Hausa and, in some cases, mixed with English.

### Data analysis

For the FGD, each session was labelled based on the category of participants and the settlement type. In contrast, for the KII, each participant’s audio recording was labelled using a unique number. The audio recordings were downloaded onto a laptop and transcribed by the RAs verbatim regardless of the interview language. Transcription of interviews conducted in Yoruba/Hausa were forward translated to English. Field notes, including each participant’s details, such as age, gender, occupation, and settlement type, were also collated. All transcribed notes were audited and validated by members of the study team.

Thematic content analysis was used to analyse the data. Relevant sections from the transcripts were extracted using the inductive-dominant coding approach [24], and preliminary codes were developed and imported into NVivo software (Version 14.0). The codes were structured, organized, reviewed, merged, and linked to the corresponding quotations. Various themes emerged and were explored in alignment with the study's objectives, focusing on key areas of consensus, contrasting perspectives, and notable as well as salient points highlighted by the participants. Verbal quotes supporting each theme were also extracted and presented in the result section. Trustworthiness was ensured by explaining detailed descriptions of the study context to participants, obtaining comprehensive daily field notes and reflexive diaries.

### Ethical considerations

Ethical approval for this study was granted by Nigeria's National Health Research Ethics Committee (Approval Number: NHREC/01/01/2007–10/10/05/2022), Health Research Ethics Committees of Oyo State Ministry of Health (Reference number: AD 13/479/44421A), Kano State Ministry of Health (Approval Number: NHREC/17/03/2018), University of Ibadan Ethics Committee (Registration number: NHREC/05/01/2008a), Northwestern University (IRB ID: STU00217380-MOD0001) and retrospectively from Loyola University Chicago (LU Number: 218531) when lead authors changed institutions. Written informed consent was obtained from all participants. The consenting process was in a language preferred by the participants. All completed informed consent forms, interview recordings, and transcripts were secured in a passworded online platform.

## Results

The findings from this study are presented in line with the study objectives. They are further described using related themes supported by quotes from the community members’ and IHCPs’ perspectives.

### Socio-demographic characteristics of participants

A total of 157 community members were involved in the FGDs (53 males and 104 females). More participants were captured in Kano (55.4%) as compared to Ibadan (44.6%). The majority were aged 40 years and above and from informal settlements. In Kano, more participants came from formal and informal settlements, while in Ibadan, more slum residents participated (18.4% vs 6.4%). Most of the participants had secondary education (43.6%), while over a tenth had no formal education (12.8%) or only primary education (12.8%). However, Ibadan had more participants with no formal education than those from Kano (12.3% vs 7.5%). Most of the participants were traders (46.4%), artisans (18.6%) or unemployed/housewives (16.4%). In Kano, more unemployed/housewives were reported compared to Ibadan (15.7% vs 0.7%).

Twelve IHCPs (7 males and 5 females) were also interviewed. Their mean (SD) age was 35 ± 4.3 years, and they had practiced for an average of 15.2 years. Half (6) of the IHCPs were from informal settlements, while the rest were evenly distributed between formal (3) and slum (3) settlements.

### Burden of malaria

The burden of malaria was consistently emphasized as high in both cities. Participants overwhelmingly identified malaria as a widespread health issue within their communities, irrespective of settlement type. They described malaria as endemic, noting that it is a persistent disease they have lived with due to its year-round occurrence. Some participants estimated that malaria accounts for about half of all childhood illnesses in their area and emphasized its severity, referring to it as deadly. Additionally, children and pregnant women were identified as the groups most vulnerable to malaria.

*“Malaria is the most common disease that is affecting everyone, especially this year, both the children and the adults, and it is deadly because we have so many patients that die due to this malaria (KII 16, Traditional Doctor, Informal, Kano)”*

Poor environmental hygiene and sanitation that promote the breeding of mosquitoes were some of the major factors identified by both community members and IHCPs as drivers of malaria in all settlement types especially in informal and slum settlements. The participants mentioned open gutters, overfilled dumpsites, and the presence of water bodies as some of the environmental problems.

*"... the cause of it is that the whole environment is polluted; many houses have gutters that go through the front and back of the house, and mosquitoes are always in the gutter, and when mosquitoes come inside the house, it will bite the children, and so many of us did not like mosquito nets, I don't like to hang that mosquito nets...”( FGD 6, Female Participant 2, Slum, Ibadan).*

However, some participants, including community members and IHCPs, had differing opinions on the causes of malaria. Notably, some individuals from informal settlements and slums believed that malaria could be caused by food preservatives, stress, and excessive exposure to sunlight.

*“...Adults also come down with malaria through diets. A lot of food we consume nowadays has high chemical concentration in them. The use of chemicals in boosting crop production is a cause and all those chemical residues get deposited in our bones, thereby causing body aches leading to malaria. (KII14 Traditional Doctor, Slum, Ibadan)”*

*“…. It is caused by stress when you are stressed, apart from that when you go out under the sun it also causes malaria. (FGD 5, Female participant, Informal Settlement, Ibadan)*

### IHCP’s perceived competence and practice

Community members and IHCPs largely view IHCPs as competent, citing personal experiences, cultural values, and positive malaria treatment outcomes. IHCPs, particularly HDS and THs, attribute their competence to spiritual beliefs in their methods. Drug peddlers are also seen as competent for providing immediate symptom relief. In slum areas, trust in PPMVs is reinforced by their dual roles as health facility staff and PPMV store operators. Below are supporting themes and quotes.

### Positive experience of community members influences perceived competence

Community members consistently return to IHCPs, including PPMVs, DPs, THs, and HDSs, due to positive treatment outcomes and satisfaction with care. This pattern was observed across both cities, regardless of settlement type.

A community member who visits a PPMV had this to say:

“*They prescribed medicine for me. I bought and took it and I felt better…… (FGD 6, Female Participant, Informal Settlement, Kano).”*

The IHCPs also recounted their experiences treating these community members, noting that patients typically recovered from the illness, with some reporting improvement in a shorter time than expected. They believed these outcomes enhanced the community's perception of their competence.

*“.... Most people that are coming to me, once I tell them that the herbs should be taken for a week or so, before three days they will have been very okay even before the dates I gave them, so they come back (KII 13, Herbal seller, Informal, Ibadan)”*.

### Spiritual beliefs and beliefs in the effectiveness of treatment method by HDSs and THs drives perceived competence

All the herbal drug sellers and traditional healers in both cities claimed to be confident in their ability to manage suspected malaria cases. They affirmed that using natural ingredients provided by God, coupled with spiritual support, enhances their ability to treat malaria effectively.

“*I am very competent, once I know the child has malaria at tender age and they brought the child to me, I know God will help me to treat such a case (KII 13, Herbal Drug Seller, Informal Settlement, Ibadan)”.*

*“...I keep saying to you that; if you bring children and pregnant women, with God's grace, I am sure I have the competence to treat them (KII 16, Traditional Healer, Informal Settlement, Kano).”*

Most herbal drug sellers and traditional healers also believed in the superior effectiveness of herbal or traditional medicine compared to orthodox medicine, which they felt provided only temporary relief. This view was also shared by some community members, especially those from informal settlements and slums.

*“...I will say that traditional medicine is more helpful than the drugs in hospital because traditional medicine cure the illness completely while the drugs given to you in the hospital will just relieve you from it for some days and you will be sick again (KII 13 Herbal drug seller, Informal Settlement, Kano)”*

### Inappropriate malaria management practices by IHCPs queries competence

Additionally, most IHCPs often do not follow the recommended treatment guidelines for malaria. The PPMVs usually treat their clients with common monotherapy anti-malarials, while some use artemisinin-based combination therapy (ACT). However, a common practice among them is to combine these anti-malarials with multivitamins and antibiotics if they perceive malaria to be severe or persistent. On the other hand, drug peddlers typically give a combination of drugs such as paracetamol, ibuprofen, and aspirin, known locally as “akapo” in the Yoruba language.

*“....We treat complicated malaria infection with antibiotics because nowadays malaria is so resistant to antimalaria. So, if you give some people antimalarial you still need to add Amoxicillin to it… (KII 11, PPMV, Formal, Ibadan)”*

*“.... We go to Alakapo (drug hawker or seller who sells and give all sorts of combination of drugs) (FGD 3, Male Participant, Slum, Ibadan).”*

The HDSs and THs typically use various items, including leaves and tree bark, which are cooked to extract the beneficial medicinal compounds. The resulting water extract is then used for treatment. Clients may be instructed to drink, inhale, or bathe with the extract. The dosage of these herbal medicines is determined based on the perceived severity of the illness by the herbal drug sellers/traditional healers.

*“…do you know neem tree? we use it, we also use mango leaves and Zumbur leaves then lemon leaves and pawpaw leaves this are what we use for the steaming while the Zumbur is in powder form these are what we use to cure malaria….”(KII 16, Herbal drug seller, Informal, Kano)*

*“Whenever they come to me for treatment and I give them a 1.5litre bottle full of herbs, they recover from the malaria by the time they use it within the first three days. (KII 16, Herbal drug seller, Slum, Ibadan)*

Furthermore, IHCPs reported that they do not confirm malaria diagnosis before they treat their clients. They rely on the clients' signs and symptoms, and based on experience, they prescribe medications. This was common to all categories of IHCPs interviewed regardless of the city or type of settlement.

*“.......It is from the patient eye that I will know, the eyes will be yellow and the hand will be pale, she will be short of blood, so when I detect such things. I will know that it is* *severe malaria, and that is how I will know what types of herbs that I will use for that person because there are some herbs that are good to enhance blood. So if we discovered that malaria is severe, we will cook more herbs with the one that will enhance the blood for the patient to drink (KII 14, Herbal Seller, Slum, Ibadan).*

### Perceived reasons for patronizing IHCP

Participants noted that community members often visit IHCPs, particularly PPMVs/DPs, because of the affordability of medicines and the option for credit or deferred payments. Many also emphasized cultural beliefs in the effectiveness of herbal remedies as a critical motivator for visiting traditional healers or herbal drug sellers. Participants also explained that these providers are often consulted when the illness is perceived as mild, with the hope of quick relief. Another major factor highlighted was the convenience and accessibility of these IHCPs within the community, especially when formal healthcare facilities are closed or hard to reach. These findings are organized according to the themes below.

### Affordability of services

Both the community members and IHCPs affirmed that the treatment provided by the IHCPs was reasonably priced, as some participants claimed that hospitals (formal health care providers) charge about 10 times more than IHCPs. Many highlighted that IHCPs provide flexible payment options, often allowing treatment to begin without full payment upfront, further enhancing their utilization. This affordability and payment flexibility were consistently reported across both cities and in all settlement types, though more participants from informal and slum settlements emphasized these points. Participants from these areas particularly appreciated the financial accessibility of IHCPs, noting that deferred payments or installment plans allow them to seek treatment they might otherwise avoid in their communities, where financial capability is low. Some participants stated the following:

*“For me, I do rush to chemist because they say" cut your coat according to your size” I do rush to chemists because with your 1,000 naira the chemist vendor will treat you well, with injection and even tablets (drugs), but when you go to hospital there you can spend more than ten thousand naira, before they treat you, they will admit you and so on which cost a lot of charge. So, for me I prefer going to the chemist (FGD 7, Female Participant Formal, Kano).”*

*“We mostly go to chemist because they can give drug when we don’t have money and can pay later (FGD 5, Female Participant, Informal, Kano).”*

*“.... if they prescribed to me and I don’t have enough money to buy everything that was prescribed, I have checked and the money is not enough, I will just go and buy the traditional ones (FGD 6, Female Participant, Slum, Kano).*

### Perceived mildness of illness

Some community members in both cities and all settlement types reported that their decision to visit IHCPs is influenced by their perception of the illness’s severity. They believe that if the symptoms of malaria are mild, there is no need to seek treatment at formal health facilities such as clinics or hospitals. Instead, they feel that such cases can be adequately managed by purchasing medication from PPMVs or seeking remedies from herbal drug sellers.

*“.... If the child’s condition is not too bad before going to the hospital, we go to the chemist for treatment (FGD 7, Mother of U5, Formal Settlement, Kano).”*

*“People go to the herb sellers as a first step at least to look at it, when it gets to two or three days, and there are no changes then they will go to the health center …. (FGD 5 Female Participant, Informal Settlement, Ibadan).”*

### Accessibility and prompt service

The IHCPs across both cities identified accessibility as one of the significant reasons community members often use their services. The IHCPs, especially PPMVs, reported they are closer to the community members and can be accessed 24 h a day, even on weekends when some primary healthcare facilities are closed. Some IHCPs also mentioned that they are frequented because community members prefer the quick service they offer. The community members can obtain all the drugs they need in a short time, unlike waiting in long queues at health facilities where they might be prescribed similar drugs available at the PPMV stores. This opinion was identical in all settlement types.

*“The main reason people seek drugs from us is because we are closer to them. Regardless of the time, they can knock on our door and request medication” (KII 11, PPMV, Informal Settlement, Kano)”*

*“If there is occurrence of malaria during weekend among those that are not rich enough and public health facilities are not opened, they usually go to chemists (FGD 7, Mother of U5, Formal Settlement, Kano).”*

*“...For some it is time. The time they will use at a private hospital cannot be compared to a health centre. That discourages them from going. Some think it wise to just go to medicine store to buy drugs since drug will still be prescribed to them at the health centre..” (KII 12, Drug peddler, Slum, Ibadan)”*

### Belief in efficacy of herbs

Findings indicate that one primary motivator for community members visiting herbal sellers or traditional healers was the solid cultural belief in the efficacy of their treatment methods, a view corroborated by the HDS and TH themselves. Several community members, particularly those in urban slums and informal settlements, strongly affirmed that herbs had been used since the time of their ancestors, and their knowledge of herbal medicine was passed down through generations. They reinforced this belief by demonstrating how to cultivate these herbs and identify useful varieties.

*“I believe in herbs. I have malaria herbs and all in one herb at home. I believe in herbs because if you use herbs, you will pass out malaria through urine (FGD 1, Male Participant, Formal Settlement, Ibadan).”*

*“...Some have a stronger belief in the efficacy of herbal mixtures. They do have it at home and even use it as a preventative measure. (KII 14. Traditional Doctor, Slum, Ibadan)*

### Contingency treatment option

This study also found that community members often seek care from formal healthcare providers and some IHCPs, such as PPMVs, for conventional treatments. Still, they frequently turn to herbal or traditional medicine when these treatments fail to meet their expectations. They noted that when conventional treatments do not provide the desired relief or are perceived as ineffective, people often shift to using herbs, believing them to offer a more lasting or holistic solution. Community members and IHCPs affirmed this practice in both cities and all settlement types.

*“I also want to say that if after taking the normal white man medicine and it did not work, it requires other native ways like herbs (FGD 7, Mother of U5, Formal, Ibadan).”*

Community members in informal and slum settlements in one of the cities also described vividly the circumstances under which they sought treatment from these categories of IHCP. As some participants said.

“…* If I took a hospital medicine, if I take it and it didn’t work for me, I have another way, and that way is I will go to a traditional doctor, our local medicines of before I will do the needful and if Allah permits, I will be fine and will recover (FGD 6, Female Participant, Informal, Kano)*

*“...We have traditional healing home like Baba Na Kabara if our children’s are sick, we try hospital first, if the drugs didn’t work for them then we go to him and buy traditional drug which is N200 to 250 naira and his drug is very effective to our children (FGD 9, Mother of U5, Slum, Kano)”*

### Perceived side effects of orthodox medicine

A few participants cited concerns about the side effects of orthodox medication as a critical reason for seeking treatment from HDS and TH. They expressed apprehension that conventional medicine often carries undesirable side effects that impact long-term health. One of the IHCPs specifically mentioned fears regarding the effects of orthodox medicine on fertility and male sexual potency, with some believing that regular use of these medications can lead to reduced fertility or diminished sexual performance in men.

*“Some of this orthodox medicine used to weaken manhood or kill cells in the body, it is the chemicals that are used to prepare those orthodox medicine that cause it, but herbs do not have such things because there are no chemicals in them (KII 14, Herbal drug seller, Slum, Ibadan).”*

## Discussion

This study explores malaria burden, IHCP competence, and patronage reasons across Nigerian urban settlements from the perspectives of the users and providers, identifying areas for improvement to integrate IHCPs into formal healthcare and enhance malaria services. Findings revealed the persistence of malaria in the studied Nigerian cities, despite the common assumption that it is primarily a rural issue and in contrast to recent findings from a quantitative study reporting low urban malaria prevalence [25]. This suggests that there is sustained malaria transmission in Nigerian cities, which is, however, in line with previous studies in the same setting where this study was conducted [26, 27].

Environmental factors that support the breeding of malaria vectors were identified as the main drivers of malaria transmission, and these were serious concerns in informal settlements and slums. This is, however, not unexpected because in informal settlements and slums, the level of hygiene is usually low, and accessibility to basic amenities such as potable water remains a challenge in sub-Saharan countries like Nigeria [28]. Household proximity to water bodies such as rivers was also identified as a driver of malaria transmission especially in informal settlements, consistent with studies in Nigeria and Africa linking higher malaria positivity rates to informal settlements and nearby water reservoirs [25, 26, 29, 30].

However, the misconception that chemicals in food and excessive sunlight are causes of malaria is worth noting. This shows that such misconceptions still exist and can impede the healthcare-seeking behaviour of community members and the management of malaria by IHCPs. Similar misconceptions have also been reported by other studies in Nigeria, thus corroborating findings from this study, necessitating public enlightenment on malaria epidemiology [31, 32].

Findings from this study suggest that IHCPs are widely patronized in the studied cities irrespective of settlement types. The competence of the IHCP was perceived as adequate from the perspective of the community members and IHCPs themselves. The community members’ perception was rooted in personal experiences and cultural beliefs, while the IHCPs attributed their competence to spiritual support. The positively perceived competence translated to the community members’ continual patronage of these IHCPs. Other researchers have also reported similar findings where one of the significant reasons for patronizing IHCPs, such as herbal drug sellers, was their belief in their competence in treating malaria cases [33].

Patent Proprietary Medicine Vendors are more widely visited in all settlement types as the first point of call, especially when symptoms appear mild. This finding is in keeping with previous studies conducted in Nigeria, which report that most of those who sought care for fever or suspected malaria visited a PPMV as the first line of treatment [15, 34].^.^ This practice highlights a common belief that professional medical care is only needed for severe illnesses, while mild symptoms can be managed through informal channels like traditional remedies or over-the-counter treatments.

Most community members and IHCPs expressed the view that the affordability of drugs at these PPMVs is also a significant factor driving people to seek care from them. They are also widely visited because they offer credit facilities (flexible payment plans), which makes it easier for individuals to access malaria treatment at the community level in all settlement types. These findings are corroborated by previous research in Nigerian cities [11, 35]. However, the lack of variation by settlement type indicates that the high cost of these drugs at healthcare facilities affect the entire city, making it difficult for the populace to access appropriate care.

The cost of treatment is a massive problem in Nigeria, probably due to the extent to which the populace depends on out-of-pocket expenditure. Strategies to reduce out-of-pocket spending, such as community-based insurance schemes and community-led health saving schemes, might improve appropriate malaria treatment [36]. Findings from this study also suggest that the same type of medications is being prescribed at both the PPMVs and formal healthcare facilities; thus, if the drugs are affordable at the healthcare facilities, the probability of high patronage would increase, and appropriate diagnosis and treatment would be guaranteed.

Herbal drug sellers and traditional healers are also well-patronized, particularly in the slums, as observed from this study. These findings align with previous research conducted in some Nigerian urban slums, where most residents believed in the efficacy of herbal drugs for treating malaria [13, 37]. Although these studies were conducted about a decade ago, and despite various interventions and programmes to improve access to proper malaria case management, it is noteworthy that HDSs are still well-patronized in present-day cities. These findings are further corroborated by Goodman et al*.* [38], whose 2022 study in an urban slum in a similar setting reported that most participants purchased herbs when experiencing symptoms suggestive of malaria, a common practice among those with low education levels. Furthermore, the HDSs and THs express confidence in their treatments, often deemed more effective than orthodox medicine, as documented in recent literature [39]. Community members emphatically stated the solid cultural belief in these herbal medications as the reason for continual patronage. This suggests a strong cultural influence on these individuals' healthcare-seeking behavior amidst issues of educational level and inaccessibility of health facilities in these urban slums. This practice could also be related to the high cost of orthodox medicine at health facilities, as some of the participants, especially in the slums, reported financial incapability as the main reason for initially visiting these HDSs and THs. These findings underscore the need to educate the inhabitants of urban slums and make healthcare affordable and accessible to them. Further research into the beneficial components of herbal medications could support their integration into formal healthcare systems. This would help bridge gaps in malaria service delivery while leveraging the cultural acceptance and accessibility of herbal remedies, ultimately enhancing malaria control efforts in underserved urban communities.

This study also examined the management practices of these IHCPs. Some PPMVs adhere to using ACT, while some prescribe monotherapy anti-malarial in combination with other drugs such as multivitamins and antibiotics. On the other hand, HDSs and THs manage malaria cases with herbal mixtures and concoctions made from tree bark, leaves, and other natural ingredients. These practices deviate from the case management guidelines for appropriate malaria treatment outlined by the WHO, which could contribute to anti-malarial drug resistance, thereby threatening successful malaria control initiatives to achieve elimination [40].

Furthermore, a common finding among all categories of IHCPs interviewed in this study is the lack of malaria diagnosis before treatment. This casts doubt on their competence and medications' proclaimed efficacy, as it is unclear if malaria is being treated. Some previous studies [14, 41] reported similar management practices, indicating that patrons of these providers often do not receive adequate malaria diagnosis before treatment despite the implementation of the WHO’s Test, Treat, and Track (T3) Initiative in Nigeria [42].

Inadequate malaria diagnosis and treatment significantly impact malaria control, leading to delayed appropriate care and increased risk of complications from fever-related conditions. Our study revealed that participants often sought other IHCPs even after receiving orthodox treatment, resulting in prolonged care pathways and higher household out-of-pocket expenditures. These delays may exacerbate the challenges of anti-malarial drug resistance and reduction of under-five mortality. Addressing this requires educating communities to request rapid diagnostic testing (RDT) before treatment and integrating IHCPs into WHO strategies to ensure universal access to diagnostic kits and appropriate treatments. Some studies in Nigeria [43–45] have documented the feasibility of integrating these IHCPs who are usually community members, by leveraging capacity-building initiatives for volunteer community health workers to enhance malaria case management at the community level, in alignment with the Global Technical Strategy for Malaria (2016–2030) [1].

### Strengths and limitations

This qualitative study has limitations, including reliance on self-reported data, which may introduce social desirability or recall bias. The cross-sectional design limits insights into changes over time, and the purposive selection of participants from specific settlements reduces the generalizability of findings to all IHCPs in urban Nigeria. Despite these constraints, a key strength of this study is that it gathers insights from users and providers perspectives, into the patronage of IHCPs for malaria treatment across various settlement types from two distinct ethnocultural cities in Nigeria— an underexplored area. These findings will significantly contribute to understanding and determining the best approach to integrate these groups of IHCPs into the already complex formal healthcare system.

## Conclusions

Malaria persists across all settlement types in the studied Nigerian cities, with poor environmental hygiene and sanitation as primary contributors in informal settlements and slums. Widespread patronage of IHCPs is driven by affordable drugs, perceived mildness of illness, credit facilities, and cultural beliefs- especially among slum residents favoring herbal drug sellers and traditional healers. Both IHCPs and their patrons viewed the IHCPs as competent in treating malaria. However, inadequate practices in malaria diagnosis and treatment were prevalent. Addressing these gaps through education, public awareness, affordable healthcare, and structured payment plans is essential to improve malaria management and facilitate IHCP integration into the formal healthcare system for effective malaria service delivery.

## Data Availability

All data were generated from the transcripts of the interviews in this qualitative study which can be made available upon request. However, most of the available data has been used as supporting quotes within the manuscript.

## Appendix I

Description of FGD sessions

**Table:**

Sn	Code	Category	No. of participants	Settlement Type	City
1	FGD 1	Male adults	9	Formal	Ibadan
2	FGD 2	Male adults	8	Informal	Ibadan
3	FGD 3	Male adults	10	Slum	Ibadan
4	FGD 4	Female adults	6	Formal	Ibadan
5	FGD 5	Female adults	7	Informal	Ibadan
6	FGD 6	Female adults	10	Slum	Ibadan
7	FGD 7	Mothers of under five	8	Formal	Ibadan
8	FGD 8	Mothers of under five	7	Informal	Ibadan
9	FGD 9	Mothers of under five	9	Slum	Ibadan
10	FGD 1	Male	10	Formal	Kano
11	FGD 2	Male	11	Informal	Kano
12	FGD 3	Male	10	Informal	Kano
13	FGD 4	Female	8	Formal	Kano
14	FGD 5	Female	10	Informal	Kano
15	FGD 6	Female	10	Informal	Kano
16	FGD 7	Mothers of under five	10	Formal	Kano
17	FGD 8	Mothers of under five	10	Informal	Kano
18	FGD 9	Mothers of under five	10	Slum	Kano

## Appendix II

Description of KII Participants

**Table:**

Sn	Code	Category of IHCP	Gender	Duration in position(years)	Settlement type	City
1	KII 11	PPMV	Female	15	Formal	Ibadan
2	KII 12	Drug peddler	Female	30	Slum	Ibadan
3	KII 13	Herbal drug seller	Female	12	Informal	Ibadan
4	KII 14	Traditional healer	Male	10	Slum	Ibadan
5	KII 15	PPMV	Female	14	Formal	Ibadan
6	KII 16	Herbal drug seller	Female	28	Slum	Ibadan
7	KII 11	PMV	Male	10	Formal	Kano
8	KII 12	Drug peddler	Male	20	Informal	Kano
9	KII 13	Herbal drug seller	Male	10	Informal	Kano
10	KII 14	Traditional healer	Male	15	Informal	Kano
11	KII 15	PPMV	Male	17	Informal	Kano
12	KII 16	Traditional healer	Male	35	Informal	Kano

## References

[1] WHO. Global technical strategy for malaria 2016–2030. Geneva, World Health Organization, 2021.

[2] WHO. World malaria report 2023. Geneva, World Health Organization. https://www.who.int/teams/global-malaria-programme/reports/world-malaria-report-2023.

[3] Odii A, Arize I, Agwu P, Mbachu C, Onwujekwe O. To what extent are informal healthcare providers in slums linked to the formal health system in providing services in sub-Sahara Africa? A 12-year scoping review. J Urban Health. 2024;101:1248–58. 38874863 10.1007/s11524-024-00885-5 PMC11652447

[4] WHO. Engaging the private health service delivery sector through governance in mixed health systems strategy report of the WHO advisory group on the governance of the private sector for universal health coverage. Geneva: World Health Organization; 2020.

[5] Bloom G, Standing H, Lucas H, Bhuiya A, Oladepo O, Peters DH. Making health markets work better for poor people: the case of informal providers. Health Policy Plan. 2011;26(Suppl 1):i45-52. 21729917 10.1093/heapol/czr025

[6] National Population Commission (NPC) [Nigeria] and ICF. 2019. Nigeria Demographic and Health Survey 2018.Abuja, Nigeria, and Rockville, Maryland, USA: NPC and ICF.

[7] Oluchi SE, Manaf RA, Ismail S, Udeani TK. Predictors of health-seeking behavior for fever cases among caregivers of under-five children in malaria-endemic area of Imo State, Nigeria. Int J Environ Res Public Health. 2019;16:3752. 31590340 10.3390/ijerph16193752 PMC6801834

[8] Vlahov D, Galea S. Urbanization, urbanicity, and health. J Urban Health. 2002;79(Suppl 1):S1–12. 12473694 10.1093/jurban/79.suppl_1.S1 PMC3456615

[9] Dovey K, Pafka E, Ristic M. Mapping urbanities: morphologies, flows, possibilities (1st edn.). Routledge. 2018.

[10] Aliyu AA, Amadu L. Urbanization, cities, and health: the challenges to Nigeria - a review. Ann Afr Med. 2017;16:149–58. 29063897 10.4103/aam.aam_1_17 PMC5676403

[11] Ogunwale A, Ajayi I, Bamgboye E, Adamu A, Bello M, Olawuwo M, et al. Are urban residents seeking the right treatment for malaria: evidence from an exploratory qualitative study in two cities in Nigeria. Research Square. 2024 (pre-print). 10.1186/s12913-024-12013-9 PMC11653962 39696418

[12] Famoyegun JD, Ogunwale AO. Malaria illness experiences and health-seeking behaviours among in-school adolescents in Ibadan North Local Government Area. South-west Nigeria Afr J Biomed Res. 2023;26:223–9.

[13] Oreagba IA, Oshikoya KA, Amachree M. Herbal medicine use among urban residents in Lagos. Nigeria BMC Complement Altern Med. 2011;11:117. 22117933 10.1186/1472-6882-11-117 PMC3252251

[14] Uchenna AA, Chinedu OA, Saleh J, Sadiqq A, Calista UN, Abonyi I, et al. Assessing the knowledge, practice and attitudes (KPA) of traditional healers in malaria control programme in Nsukka Zone of Enugu State Nigeria. J Health Med Nurs. 2018;49:37–49.

[15] Uzochukwu BSC, Ossai EN, Okeke CC, Ndu AC, Onwujekwe OE. Malaria knowledge and treatment practices in Enugu State, Nigeria: a qualitative study. Int J Health Policy Manag. 2018;7:859–66. 30316234 10.15171/ijhpm.2018.41 PMC6186483

[16] Sudhinaraset M, Ingram M, Lofthouse HK, Montagu D. What is the role of informal healthcare providers in developing countries? A systematic review PLoS One. 2013;8: e54978. 23405101 10.1371/journal.pone.0054978 PMC3566158

[17] Alyafei A, Easton-Carr R. The health belief model of behavior change. In: StatPearls Publishing; 2024. Available from: https://www.ncbi.nlm.nih.gov/books/NBK606120/39163427

[18] Dougherty L, Gilroy K, Olayemi A, Ogesanmola O, Ogaga F, Nweze C. Understanding factors influencing care seeking for sick children in Ebonyi and Kogi States. Nigeria BMC Public Health. 2020;20:746. 32448259 10.1186/s12889-020-08536-5 PMC7245913

[19] Jombo GTA, Mbaawuaga EM, Akaa PD, Dauda AM, Eyong KI, Akosu JT, et al. Utilization of traditional healers for treatment of malaria among female residents in Makurdi city and its environs. Asian Pacific J Trop Med. 2010;3:563–6.

[20] Uneke CJ, Obeka I, Uneke BI, Umeokonkwo A, Nweze CA, Otubo KI, et al. An assessment of nursing mothers’ and young people’s access to proprietary and patent medicine vendors’ services in rural communities of south-eastern Nigeria: implication for review of national drug policy. J Pharm Policy Pract. 2021;14(Suppl 1):92. 34784979 10.1186/s40545-021-00334-7 PMC8594071

[21] WorldPop Nigeria Population Raster Data**.** (2024). *WorldPop.* Retrieved September 2024 from https://wopr.worldpop.org/NGA/

[22] GRID3 Nigeria Geospatial Data. (2024). *GRID3: Geospatial Resources for Development.* Retrieved September 2024 from https://grid3.org/geospatial-data-nigeria

[23] Ozodiegwu ID, Ogunwale AO, Surakat O, Akinyemi JO, Bamgboye EA, Fagbamigbe A, et al. Description of the design of a mixed-methods study to assess the burden and determinants of malaria transmission for tailoring of interventions (microstratification) in Ibadan and Kano metropolis. Malar J. 2023;22:255. 37661263 10.1186/s12936-023-04684-2 PMC10476435

[24] Armat M, Assarroudi A, Rad M, Sharifi H, Heydari A. Inductive and deductive: ambiguous labels in qualitative content analysis. Qualitative Report. 2018;23:219–21.

[25] Chiziba C, Mercer LD, Diallo O, Bertozzi-Villa A, Weiss DJ, Gerardin J, et al. Socioeconomic, demographic, and environmental factors may inform malaria intervention prioritization in urban Nigeria. Int J Environ Res Public Health. 2024;21:78. 38248543 10.3390/ijerph21010078 PMC10815685

[26] Awosolu OB, Yahaya ZS, Farah Haziqah MT, Simon-Oke IA, Fakunle C. A cross-sectional study of the prevalence, density, and risk factors associated with malaria transmission in urban communities of Ibadan, Southwestern Nigeria. Heliyon. 2021;7: e05975. 33521357 10.1016/j.heliyon.2021.e05975 PMC7820925

[27] Olukosi AY, Olakiigbe A, Ajibaye O, Orok BA, Aina OO, Akindele SK, et al. Socio-economic behavioural indicators of falciparum malaria parasitaemia and moderate to severe anaemia among pregnant women attending antenatal clinics in Lagos, Southwest Nigeria. Malar J. 2020;19:393. 33160357 10.1186/s12936-020-03462-8 PMC7648425

[28] Hassan AW, Zhang D, Ibrahim M. Accessibility to WASH and waste management services in African urban informal settlements: a comparative analysis. J Water Sanit Hyg Develop. 2024;14:91–112.

[29] Hassen J, Dinka H. Magnitude of urban malaria and its associated risk factors: the case of Batu town, Oromia Regional State, Ethiopia. J Int Med Res. 2022;50:03000605221080686. 35259963 10.1177/03000605221080686 PMC8918979

[30] Ugwu CLJ, Zewotir T. Evaluating the effects of climate and environmental factors on under-5 children malaria spatial distribution using generalized additive models (GAMs). J Epidemiol Glob Health. 2020;10:304–14. 33009733 10.2991/jegh.k.200814.001 PMC7758859

[31] Nwogu JN, Igbolekwu CO, Nwogu E, Nwokocha EE, Ogadimma AC, Ekechukwu VI. Perception and experience of childhood malaria management among mothers of under-five children in Osogbo Osun State, Nigeria. Research Square; 2021 (pre-print).

[32] Tobin-West CI, Kanu EN. Factors influencing the use of malaria prevention methods among women of reproductive age in peri-urban communities of Port Harcourt city Nigeria. Niger Postgrad Med J. 2016;23:6–11. 27098942 10.4103/1117-1936.180114

[33] Afriyie J, Kumi-Kyereme A. Predictors of herbal medicine use in Ashanti Region of Ghana. Adv Integrat Med. 2023;10:80–5.

[34] Prach LM, Treleaven E, Isiguzo C, Liu J. Care-seeking at patent and proprietary medicine vendors in Nigeria Health systems and services in low- and middle-income settings. BMC Health Serv Res. 2015;15:231. 26067426 10.1186/s12913-015-0895-z PMC4465150

[35] Millar KR, McCutcheon J, Coakley EH, Brieger W, Ibrahim MA, Mohammed Z, et al. Patterns and predictors of malaria care-seeking, diagnostic testing, and artemisinin-based combination therapy for children under five with fever in Northern Nigeria: a cross-sectional study. Malar J. 2014;13:447. 25413231 10.1186/1475-2875-13-447 PMC4253990

[36] Effiong FB, Ogbonna CP, Agughalam PI, Okwukwu MO, Dike IC, Elebesunu EE, et al. The role of community-based approaches in achieving universal health coverage: addressing the Nigerian narrative. Ann Med Surg (Lond). 2023;85:1769–73. 37228928 10.1097/MS9.0000000000000443 PMC10205343

[37] Olorunniyi OF, Morenikeji OA. The extent of use of herbal medicine in malaria management in Ido/Osi local government area of Ekiti State. Nigeria J Med Plants Res. 2013;7:3171–8.

[38] Goodman OO, Adejoh SO, Adeniran A, Emechebe AC, Kuyinu YA. We love orthodox medicine but still use our “Elewe omo”: utilization of traditional healers among women in an urban community in Nigeria. J Family Med Prim Care. 2022;11:215–23. 35309609 10.4103/jfmpc.jfmpc_1302_21 PMC8930113

[39] Ochepo P, Ali N, Farrant A. The views of traditional healers on health-seeking behaviour for malaria treatment: a qualitative study in Makurdi, Nigeria. Divers Equal Health Care. 2022;19:51.

[40] WHO. Guidelines for malaria, 2023. Geneva, World Health Organization, 2023.

[41] Isiguzo C, Anyanti J, Ujuju C, Nwokolo E, De La Cruz A, Schatzkin E, Modrek S, et al. Presumptive treatment of malaria from formal and informal drug vendors in Nigeria. PLoS ONE. 2014;9: e110361. 25333909 10.1371/journal.pone.0110361 PMC4204870

[42] WHO. T3: Test, Treat, Track Brochure. Geneva, World Health Organization, 2012.

[43] Ajayi IO, Nsungwa-Sabiiti J, Siribié M, Falade CO, Sermé L, Balyeku A, et al. Feasibility of malaria diagnosis and management in Burkina Faso, Nigeria, and Uganda: a community-based observational study. Clin Infect Dis. 2016;63(Suppl 5):S245–55. 27941101 10.1093/cid/ciw622 PMC5146694

[44] Castellani J, Nsungwa-Sabiiti J, Mihaylova B, Ajayi IO, Siribié M, Afonne C, et al. Impact of improving community-based access to malaria diagnosis and treatment on household costs. Clin Infect Dis. 2016;63(Suppl 5):S256–63. 27941102 10.1093/cid/ciw623 PMC5146695

[45] Adeoti O, Spiegelman D, Afonne C, Falade CO, Jegede AS, Oshiname FO, et al. The fidelity of implementation of recommended care for children with malaria by community health workers in Nigeria. Implement Sci. 2020;15:13. 32131852 10.1186/s13012-020-0968-1 PMC7057616

