# Correction: Understanding malaria treatment patronage from informal healthcare providers in Nigerian urban settlements: insights from community members and providers

**DOI:** 10.1186/s12936-025-05328-3

**Published:** 2025-04-24

**Authors:** Eniola A. Bamgboye, Akintayo Olamide Ogunwale, Adamu Al-Mukhtar, Bello Musa, Laurette Mhlanga, Morenikeji Olawuwo, Adeniyi Fagbamigbe, Joshua Akinyemi, IkeOluwapo Ajayi, Ifeoma D. Ozodiegwu

**Affiliations:** 1 Department of Health Informatics and Data Science, Loyola University Chicago, Chicago, IL USA; 2 Epidemiology and Biostatistics Research Unit, Institute for Advanced Medical Research and Training (IAMRAT), College of Medicine, University of Ibadan, Ibadan, Nigeria; 3 Department of Public Health, Bowen University, Iwo, Nigeria; 4 Department of Medical Microbiology and Parasitology, Bayero University, Kano, Nigeria; 5 Department of Community Medicine, Bayero University, Kano, Nigeria; 6 Department of Epidemiology and Medical Statistics, University of Ibadan, Ibadan, Nigeria

**Correction: Malaria Journal (2025) 24:26** 10.1186/s12936-025-05255-3

Following publication of the original article [1], the authors flagged an error in the Conclusions of the Abstract: where it now (correctly) says ‘IHCPs remain consistently patronized across urban settlements.’ it (incorrectly) said ‘IHCPs remain consistently patronized across urban settlements. IHCPs are continuously patronized in all urban settlement’. The authors thank you for reading this erratum and apologize for any inconvenience caused.
